# Interactive effects of culture and sex hormones on the sex role self-concept

**DOI:** 10.3389/fnins.2015.00240

**Published:** 2015-07-14

**Authors:** Belinda Pletzer, Ourania Petasis, Tuulia M. Ortner, Larry Cahill

**Affiliations:** ^1^Department of Psychology, University of SalzburgSalzburg, Austria; ^2^Centre for Cognitive Neuroscience, University of SalzburgSalzburg, Austria; ^3^Department of Neurobiology and Behavior, University of CaliforniaCalifornia, CA, USA

**Keywords:** sex role, menstrual cycle, hormonal contraceptives, sex hormones

## Abstract

Sex role orientation, i.e., a person's masculinity or femininity, influences cognitive and emotional performance, like biological sex. While it is now widely accepted that sex differences are modulated by the hormonal status of female participants (menstrual cycle, hormonal contraceptive use), the question, whether hormonal status and sex hormones also modulate participants sex role orientation has hardly been addressed previously. The present study assessed sex role orientation and hormonal status as well as sex hormone levels in three samples of participants from two different cultures (Northern American, Middle European). Menstrual cycle phase did not affect participant's masculinity or femininity, but had a significant impact on reference group. While women in their follicular phase (low levels of female sex hormones) determined their masculinity and femininity in reference to men, women in their luteal phase (high levels of female sex hormones) determined their masculinity and femininity in reference to women. Hormonal contraceptive users rated themselves as significantly more feminine and less masculine than naturally cycling women. Furthermore, the impact of biological sex on the factorial structure of sex role orientation as well as the relationship of estrogen to masculinity/femininity was modulated by culture. We conclude that culture and sex hormones interactively affect sex role orientation and hormonal status of participants should be controlled for when assessing masculinity and/or femininity.

## Introduction

*Sex role orientation*, also referred to as *gender role orientation, gender role identity, gender role self-concept*, or *gender-related self* has been described as a person's identification with personal attributes that are seen as appropriate for a typical man or woman in a given society, i.e., his or her degree of maleness or femaleness (e.g., Sieverding et al., 2005). These attributes have revealed to be related to various behaviors in numerous psychological domains, as, for example, health (Lefkowitz and Zeldow, 2006; Zimmermann et al., 2011), occupation and work (Sieverding et al., 2005; Garcia-Retamero and Lopez-Zafra, 2009), education (Ritter, 2004; Wolfram et al., 2009; Kessels and Steinmayr, 2013) and cognitive performance (REFs). Some studies demonstrate that sex role orientation was even more influential on outcome measures than biological sex (e.g., Cahill et al., 2004). Consequently, individual differences in sex role orientation, have attracted considerable interest in psychological research since Bem's seminal work (Bem, 1974).

During the past decades it has come into focus, that sex differences are modulated by sex hormone levels and consequently, the hormonal status of female participants, i.e., menstrual cycle phase as well as hormonal contraceptive use. Abilities typically stronger in men than women, like e.g., spatial abilities, are more pronounced in naturally cycling women during their low-hormone follicular phase (Hampson, 1990; Hausmann et al., 2000; McCormick and Teillon, 2001; Dadin et al., 2009). On the contrary, abilities typically stronger in women than in men, like verbal and memory performance are more pronounced in naturally cycling women during their high-hormone luteal phase (Hampson, 1990; Rosenberg and Park, 2002; Dadin et al., 2009). Likewise, hormonal contraceptives display both masculinizing, e.g., enhanced spatial abilities (Wright and Badia, 1999; Wharton et al., 2008), and feminizing, e.g., enhanced verbal and memory abilities (Wright and Badia, 1999; Mordecai et al., 2008), effects on cognitive performance. Furthermore, spatial abilities have been repeatedly related to estradiol and testosterone levels, although there is some discourse about the specific nature of the relationship (e.g., Tan, 2012), while results on the impact of sex hormone levels on verbal- and memory abilities are still inconsistent (e.g., Andreano and Cahill, 2009).

However, studies directly relating sex hormone levels to sex role orientation are rare—mostly focusing on adult and prenatal testosterone (Baucom et al., 1985; Udry, 2000; Al-Ayadhi, 2004), while the question as to whether hormonal status also affects sex role orientation has not been addressed previously. Do women during their follicular phase also perceive themselves as more masculine compared to women during their luteal phase? Do women on hormonal contraceptives actually perceive themselves as more masculine or feminine than naturally cycling women?

These questions tap into the old debate, whether a person's maleness or femaleness, like any other personality characteristics, is determined by socialization or vice versa to which extent it is determined by genetics or other biological factors, like sex hormone levels. With reference to the social role theory, gender roles reflect the traditional social roles of male breadwinners and female caregivers (Eagly and Steffen, 1984; Bosak et al., 2012; Wood and Eagly, 2012). On the other hand, research indicating a role of prenatal androgen exposure for the development of gender-typical behavior endorsed biologically oriented theories (Pasterski et al., 2005; Rammsayer and Troche, 2007; Neave and O'Connor, 2009). At the present, biosocial interaction theories acknowledging causal roles for both biological and social influences on gender related behavior are proposed as more influential (Halpern and Tan, 2001; Eagly and Wood, 2013, p. 1). While the attributes viewed as typical for a man or woman may differ between societies and change over time, genetic factors and sex hormone levels may determine how strongly a person is able to identify herself with those roles. Consequently *gender role stress*, i.e., the amount of stress resulting from the perceived failure to meet gender role standards, has been suggested as one approach to assess gender role identification (Copenhaver and Eisler, 1996).

A number of questionnaires have been developed in order to assess sex role orientation, including the Personal Attributes Questionnaire (PAQ; Spence et al., 1974) and the Bem Sex Role Inventory (BSRI; Bem, 1974). Both questionnaires assess sex role orientation by means of self-ascribed personality characteristics including mainly socially desirable and stereotypical self-perceived personality traits (see Lenney, 1991). These characteristics are assigned to distinct scales for masculinity and femininity. Thus, masculinity and femininity are viewed as different factors (two-component model), rather than viewing the concept of sex role orientation as one bipolar dimension with masculinity and femininity presenting opposite poles of the same concept. While a typical male integrates mostly masculine features and a typical female mostly feminine features, two additional sex-types have been derived from this two-dimensional approach. “*Androgyny*” (Greek: ανήρ, stem ανδρ- *anér-/andr-* = man; γυνή *gyné* = woman) refers to the integration of both masculine and feminine identifications into a person's self-concept, while “*indifference*” refers to the lack of both masculine and feminine identifications. About a third of subjects from Bem's original study display androgyny (Bem, 1974).

Besides the fact that information gained by all self-report measures of gender roles may be biased through introspective limits or strategies of self-presentation (see also van Well et al., 2007), research on the most frequently used measure, the BSRI, revealed several additional flaws. Based on intercultural data it was shown that the questionnaire items did not share its psychometric properties throughout cultures (Sugihara and Katsurada, 1999; Peng, 2006; Colley et al., 2009), and not throughout time (Ballard-Reisch and Elton, 1992). General criticism regarding its proposed factorial structure was raised in a meta-analysis (Choi and Fuqua, 2003). While some factor analyses confirm the validity of the two-dimensional approach after excluding some of the items (Gaudreau, 1977; Waters et al., 1977), others criticize that dimensions differ between men and women (e.g., Pedhazur and Tetenbaum, 1979). Further, it was proposed that the questionnaire may exclude important aspects of gender role, as more aspects than personality traits are linked to gender (Deaux and Lewis, 1984; Athenstaedt, 2003), as, for example, abilities, relationships, physical characteristics, or occupational characteristics (Twenge, 1999).

The present study seeks to address the interplay of societal factors and sex hormone levels in a person's sex role identity and work toward a more universal measure of sex role orientation. Our specific aims are to clarify the following basic questions regarding sex role in a cross-cultural approach:

- Does culture or the hormonal status (menstrual cycle, OC use) of female participants affect their sex role orientation or whether they compare themselves to men or women in their sex role ratings?
- Is sex role orientation influenced by sex hormone levels, and if so, do sex hormones influence sex role orientation differently in men and women of different hormonal status?
- Is the two-component model (masculinity and femininity as separate factors) an adequate description of sex role orientation in men and women of different hormonal status or is the factorial structure of sex role orientation influenced by sex and hormonal status?
- According to what features do participants determine their sex role orientation in different cultures?

## Methods

### Participants

Data were collected from three samples of participants: (a) an English-speaking Northern American sample of 102 undergraduate students at the University of California, Irvine, comprised of 37 men, 40 naturally cycling women and 35 oral contraceptive (OC) users. (b) A German-speaking Middle European sample of 215 undergraduate students at the University of Salzburg, Austria, comprised of 95 men 67 naturally cycling women and 53 OC users. (c) A German-speaking Middle European Online sample of 308 adult volunteers from the general population, comprised of 73 men, 59 naturally cycling women, 82 OC users and an additional 94 women, who didn't provide us with any information on their hormonal status. The latter were excluded from further analyses. Age of participants is summarized in Table 1, cycle data of the naturally cycling groups are summarized in Table 2. All participants from samples (a) and (b) were students and had all passed their A-levels (Abitur). Sample (c) was included to also get a picture of sex role orientation from a more general population, which is less homogenous in age and socio-economic status. Although all participants from sample (c) had also passed their A-levels (Abitur), 47% of the men and 36% of the women in the sample were not University students, but in a current working relationship. Only subjects not currently on medication and without psychological, neurological, or endocrine disorders were allowed to participate.

**Table 1:** **Age [*****M*** **±** ***SD*** **(years)] of men and women in the three samples**.

	**Men**	**Naturally cycling women**	**OC users**
(a) California – PP	21.19 ± 3.13	20.30 ± 1.73	19.96 ± 1.81
(b) Austria – PP	24.56 ± 4.36	23.27 ± 3.80	21.72 ± 3.30
(c) Austria – Online	26.71 ± 10.16	24.63 ± 6.22	22.44 ± 3.35

*OC, oral contraceptive; PP, paper and pencil*.

**Table 2:** **Cycle data of the naturally cycling groups among the three samples**.

	**Cycle length**	**Follicular**		**Luteal**	
	**days (*M* ± *SD*)**	***N***	**day (*M* ± *SD*)**	***N***	**day (*M* ± *SD*)**
(a) California – PP	29.19 ± 3.52	18	7.89 ± 4.27	22	25.59 ± 6.34
(b) Austria – PP	29.72 ± 3.62	36	7.36 ± 3.71	31	24.26 ± 7.91
(c) Austria – Online	29.03 ± 3.22	31	7.06 ± 4.38	28	23.11 ± 4.65

Only naturally cycling women with a cycle length between 20 and 36 days and a variability in cycle length of no more than 7 days were included. These criteria were based on the observations of Fehring et al. (2006). Cycle phase was determined using participants self-reports of their last period date, average cycle length, and—if available—confirmed by follow-up reports of the actual onset of their next period and their estrogen and progesterone levels (see below). Participants were assigned to the early follicular groups with cycle days up to 3 days before ovulation. Participants were assigned to the luteal groups with cycle days from 2 days after ovulation to 2 days before the expected onset of their next period. Ovulation was assumed 14 days before the onset of next period.

### Ethics statement

All participants of samples (a) and (b) gave their signed written consent to participate in the study and approval by the local ethical boards was requested if required [sample (a)]. Sample (c) was an Online sample, i.e., the link to the online questionnaire was sent out to a large number of people, who were free to choose whether to participate by opening the link or not to participate. Participants could terminate the online survey at any time. Only data of those participants who fully completed the online survey were included in the analyses. All methods conform to the Code of Ethics of the World Medical Association (Declaration of Helsinki). The study on sample (a) was approved by the University of California, Irvine's Institutional Review Board. Studies on samples (b) and (c) did not require ethical approval according to the institutional guidelines of the University of Salzburg (Statutes of the University of Salzburg—see https://online.uni-salzburg.at/plus_online/wbMitteilungsblaetter.display?pNr=98160). It is stated in § 163 (1) that ethical approval is necessary for research on human subjects if it affects the physical or psychological integrity, the right for privacy or other important rights or interests of the subjects or their dependents. In § 163 (2) it is stated that it is the responsibility of the PI to decide, whether (1) applies to a study or not. Therefore, we did not approach our institutional review board to obtain ethical approval or a waiver for studies on sample (b) and (c). Since it was non-invasive and performed on healthy adult volunteers who gave their informed consent to participate, (1) did not apply.

### Sex role

**Figure 1:**
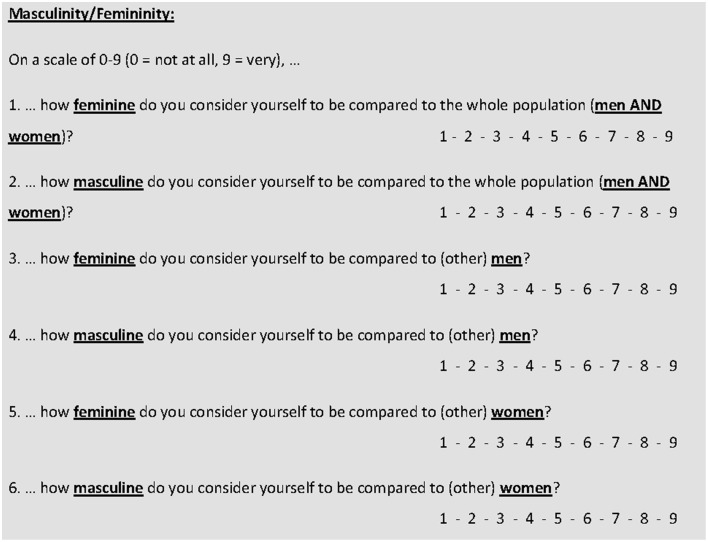
**Six item sex role scale (English version)**.

Sex role was assessed by a simple six item scale. Rather than using a detailed questionnaire of personality or other characteristics that are viewed as typically masculine or feminine in a given culture, participants directly rated their masculinity and femininity, respectively on a scale of 1–9 (Figure 1). While this approach has the disadvantage of only representing the dimensions of masculinity and femininity by a single item, it has several advantages. First, the scale can be used in different cultures irrespective of what is perceived as typically masculine or feminine in a given culture. Second, in contrast to a longer questionnaire, the small number of items allows to address differences in how participants perceive themselves not only with respect to the entire population, but also relative to the own and relative to the opposite sex. Thus, masculinity and femininity are each rated 3 times, relative to (other) men, (other) women or the entire population. Third, due to the direct approach and the short duration, the scale can be combined with a qualitative approach by giving participants the opportunity to explain their choices. These explanations can be particularly valuable to assess what is perceived as typically male or female in different cultures and this knowledge can in the long term be used to construct a more comprehensive questionnaire of sex role orientation than is currently available.

Sample (a) completed the English version of the scale, samples (b) and (c) the German version. Samples (a) and (b) completed the scale in a paper- and pencil (PP) design prior to different further experiments, which will not be reported in this manuscript. Sample (c) completed the scale as part of an online questionnaire. Therefore, sample (a) will be referred to as “California – PP,” sample (b) as “Austria – PP,” sample (c) as “Austria – Online.”

Comparable to Bem's (1974) typology, we defined participants with (i) low masculinity as well as low femininity ratings compared to the entire population as *indifferent*, (ii) high masculinity, but low femininity ratings compared to the entire population as *male*, (iii) low masculinity, but high femininity ratings compared to the entire population as *female*, and (iv) high masculinity as well as high femininity ratings compared to the entire population as *androgynous*. Rating larger than five were considered high, ratings lower than or equal to five were considered low. Female participants with a male typology and male participants with a female typology are referred to as *flips*.

In samples (a) and (c) participants were given the opportunity to give a short written explanation for their choice of sex role ratings in their own wording in order to determine the characteristics of their sex role self-concept. This was unfortunately not possible in sample (b) due to time restrictions, which is another reason why sample (c) was additionally included in the study. Reactance statements and circular references to the terms “masculine” or “feminine” were discarded. All other explanations were categorized to one of the following categories by two raters independently:

- *Face and Body:* includes statements on their genetically determined bodily appearance, e.g., muscles, body hair, breasts, and facial features.
- *Beauty:* includes statements on the value of make-up, hair styling, fashion, weight control, body building, or any other measure taken to increase one's attractiveness.
- *Activities:* includes statements on activities or interests, participants consider to be typically chosen by men or women, like sports, particular movies, shopping, meeting friends etc.
- *Social contacts:* includes statements on the proportion of males and females in the actual and/or childhood social environment of participants (parents, siblings, friends, coworkers, and other peer relations) as well as how comfortable they feel among men or women.
- *Personality:* includes statements on personality traits, participants consider as typically male or female.
- *Emotion:* includes statements on how reflective and open participants are about their emotions and whether or not they like to think or talk about their and other's emotions.
- *Cognition*: includes statements on cognitive skills, participants consider as typically male or female, e.g., orientation in a spatial environment, logics, technology, multi-tasking.

### Sex hormones

Sex hormone levels were quantified from saliva samples in samples (a) and men and naturally cycling women of sample (b) using Salimetrics and DeMediTec ELISA kits, respectively, for Progesterone, 17β-Estradiol, and Testosterone. In men and naturally cycling women all three hormones were evaluated. In OC-users only 17β-Estradiol and Testosterone were evaluated due to the high variability in synthetic progestins used in OC. Until hormone assessment, saliva samples were stored at −20°C and centrifuged two times at 3000 rpm for 15 and 10 min, respectively. For each participant we calculated the mean level for each hormone over the values assessed in the three samples that were collected over the course of an hour. The value for each sample was determined as the mean over duplicate measurements to ensure reliability of the assessment. Hormone levels with a Coefficient of Variance higher than 25 between duplicate samples were excluded. All hormone levels were within the expected range for the respective participant group.

### Statistical analyses

Statistical analyses were performed using PASW statistics 17.0. Sex role ratings were not expected to be normally distributed over all participants or in any subgroup^1^, as of course men were expected to have primarily high masculinity ratings and women were expected to have primarily high femininity ratings. Therefore, non-parametric tests were applied.

To determine, whether the hormonal status of participants affected their sex role ratings, the following analyses were performed. Mann–Whitney *U*-tests were used to compare (i) the sex role ratings of follicular and luteal women, in order to address menstrual cycle-dependent on the one hand and (ii) the sex role ratings of OC users and naturally cycling women to address oral contraceptive-dependent effects on the other hand (see Section Does Culture or Hormonal Status Affect the Sex Role Self-concept? for the results).

To determine, whether the reference group influenced participants sex role ratings, i.e., whether sex role ratings differed depending on whether they were given in comparison to men, women or the entire population, the following analyses were performed (see Section Which Participants Compare themselves more Strongly to Men, Which to Women? for the results):

- Masculinity/femininity ratings in reference to men were compared to masculinity/femininity ratings in reference to women using Wilcoxon signed ranks tests.
- Masculinity/femininity ratings in reference to the entire population were compared to the mean of masculinity/femininity ratings in reference to men and women using Wilcoxon signed ranks tests.

Additionally, to determine, whether men, naturally cycling women and OC users in the different samples compared themselves more strongly to men or women, we used a stepwise multiple regression procedure. Masculinity/femininity ratings in reference to the entire population were entered as dependent variable and masculinity/femininity ratings in reference to men and women were entered as independent variables, to see whether participants overall rating of their masculinity/femininity (i.e., their rating relative to the entire population) depended more strongly on how they perceived themselves relative to men or how they perceived themselves relative to women.

To assess whether sex hormone levels related to sex role ratings, interrelations between the six sex role ratings and the levels of testosterone, estrogen and progesterone were evaluated by Spearman correlations (i) over all participants, (ii) separately for men, follicular women, luteal women, and OC-users (see Section Does the Sex Role Self-concept Relate to Sex Hormone Levels? for the results).

To determine whether the concept of sex role orientation had a one-factorial structure with masculinity and femininity forming opposite poles of one factor or a two-factorial structure with masculinity and femininity loading high on different factors, a principal component analysis was performed using varimax rotation (see Section Do Culture, Sex, or Hormonal Status Affect the Factorial Structure of the Sex Role Self-concept for the results). Due to the small number of factors and observed variables, we did not perform a confirmatory factor analysis.

After examining the factorial structure of sex role, we explore sex role typology among men and women from different cultures by reporting the frequencies of the four typologies described above separately for men, follicular women, luteal women and OC users for each sample and comparing them between groups using χ^2^-tests (see Section Typology for the results).

The frequencies of explanations falling into the categories listed above are reported separately for men, follicular women, luteal women, and OC users as well as all participants for each sample and compared between groups and samples using χ^2^ tests (see Section Characteristics of the Sex Role Self-concept for the results).

If not specified differently, the sample sizes included in the analyses equaled the description in the participants section.

## Results

### Does culture or hormonal status affect the sex role self-concept?

#### Culture dependent effects

We did not observe any differences in masculinity and femininity ratings of men and women between the three samples in Kruskall–Wallis tests (all χ^2^ < 3.56, all *p* > 0.16).

#### Sex dependent effects

**Figure 2:**
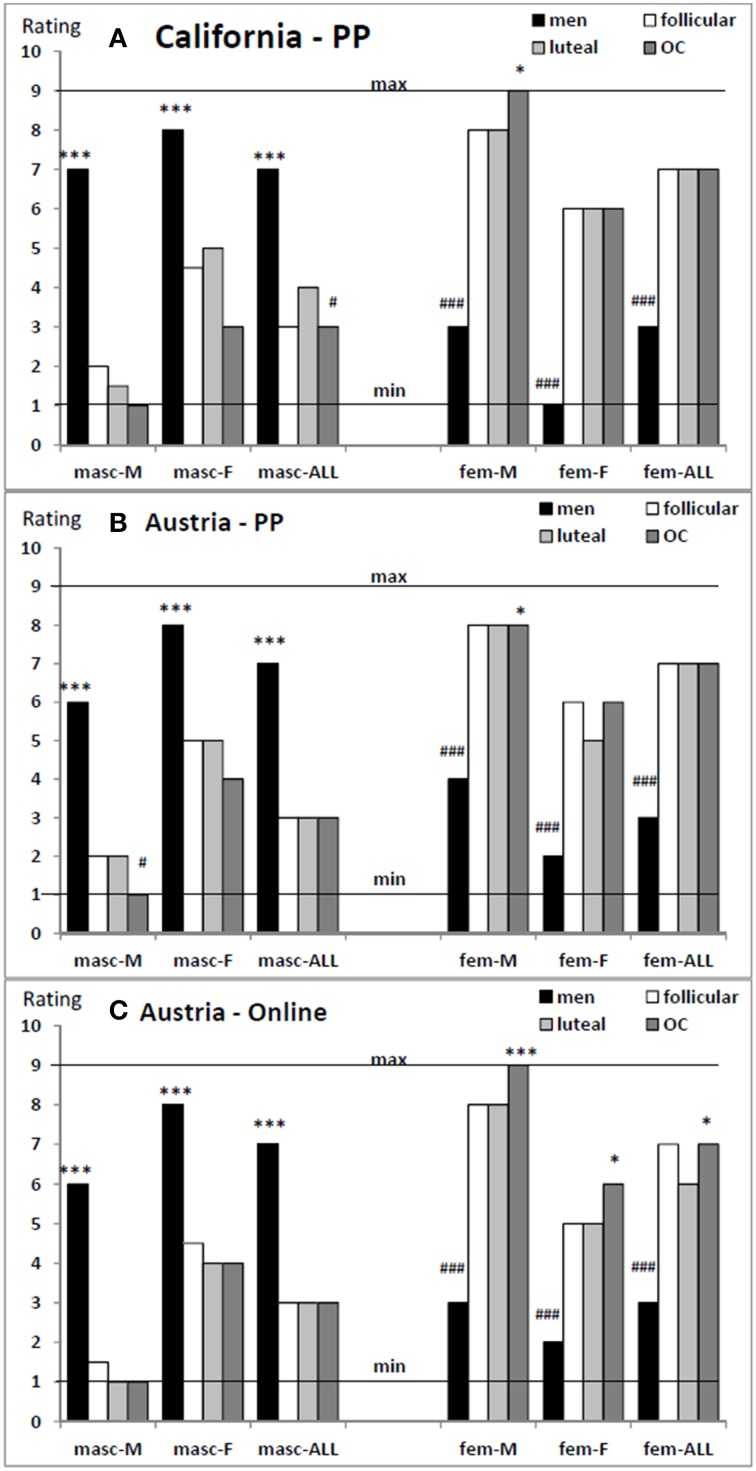
**Median of the six sex role ratings in men, follicular women, luteal women and OC users of the three samples**. Men rate themselves more masculine and less feminine than women. Follicular and luteal women did not differ in their ratings. OC-users rate themselves more feminine less masculine than naturally cycling women. All groups rate themselves more masculine and less feminine in reference to women than in reference to men. Ratings in reference to the entire population do not equal the mean of ratings in reference to men and women. PP, paper-and-pencil; OC, oral contraceptive; masc, masculine; fem, feminine; M, in reference to men; F, in reference to women; ALL, in reference to the entire population; min, minimum rating; max, maximum rating. Ratings significantly higher than in the other groups: ^*^all *p* < 0.05, ^**^all *p* < 0.01, ^***^all *p* < 0.001; ratings significantly lower than in the other groups: ^#^all *p* < 0.05, ^##^all *p* < 0.01, ^###^all *p* < 0.001. **(A)** California – PP, **(B)** Austria – PP, **(C)** Austria – Online.

As expected, in all samples men rated themselves as significantly more masculine and significantly less feminine than women irrespective of the reference group as assessed by Mann–Whitney *U*-Tests (all *Z* > 7.19, all *p* < 0.001, compare Figure 2).

#### Menstrual cycle dependent effects

We did not observe any differences between sex role ratings of follicular and luteal women in any sample (all *Z* < 1.70, all *p* > 0.08, compare Figure 2).

#### OC dependent effects

In all three samples, OC users rated themselves significantly more feminine in reference to men than naturally cycling women (all *Z* > 1.92, all *p* < 0.05). In the Austrian Online-sample drawn from the general population, all three femininity ratings were higher in OC users than in naturally cycling women (all *Z* > 2.35, all *p* < 0.05, compare Figure 2). In the California PP undergraduate sample, OC users furthermore, reached lower masculinity ratings than naturally cycling women in reference to the entire population (*Z* = 2.02, *p* < 0.05), while in the Austrian PP undergraduate sample, OC users reached lower masculinity ratings than naturally cycling women in reference to men (*Z* = 2.26, *p* < 0.05).

### Which participants compare themselves more strongly to men, which to women?

In all samples all participants rated themselves significantly more masculine in reference to women than in reference to men and significantly more feminine in reference to men than in reference to women (all *Z* > 1.95, all *p* < 0.05, compare Figure 2). We observed that in several cases the averaged ratings in reference to men and women deviated significantly from the ratings in reference to the entire population (e.g., men: all *Z* > 1.95, all *p* < 0.05). This observation raised the question, which reference group men and women of different hormonal status use to determine their ratings in reference to the entire population, i.e., which rating (in reference to men or women) has a stronger predictive value for the ratings in reference to the entire population.

Results of stepwise multiple regression are summarized in Table 3. In all three samples men determined their sex role largely in reference to men, i.e., they compared themselves more strongly to men than to women. In both undergraduate samples luteal women and OC-users determined their sex role in reference to women, i.e., they compared themselves more strongly to women than to men. However, follicular women determined their sex role largely in reference to men. In the Austrian Online sample drawn from the general population all female groups determined their sex role in reference to women.

**Table 3:** **Results of stepwise multiple regression for men, naturally cycling women and oral contraceptive (OC) users in the three samples**.

	**Men**			**Follicular**			**Luteal**			**OC**		
		**β**	***t***		**β**	***t***		**β**	***t***		**β**	***t***
**CALIFORNIA – PP**												
MascM^a^	ENT	0.66	5.16^***^	ENT	0.81	5.49^***^	REM	0.04	0.24	E/R	0.46	3.80^**^
MascF^a^	REM	0.28	1.94	REM	0.34	1.94	ENT	0.78	5.65^***^	ENT	0.82	6.96^***^
FemM^b^	ENT	0.82	8.42^***^	ENT	0.78	4.99^***^	REM	−0.07	−0.35	REM	−0.04	−0.25
FemF^b^	REM	0.20	1.53	REM	0.32	1.72	ENT	0.87	8.06^***^	ENT	0.78	6.03^***^
**AUSTRIA – PP**												
MascM^a^	ENT	0.75	11.04^***^	ENT	0.69	5.57^***^	R/E	0.32	2.47^*^	REM	0.13	0.92
MascF^a^	R/E	0.32	4.70^***^	R/E	0.47	4.11^***^	ENT	0.75	6.09^***^	ENT	0.68	5.95^***^
FemM^b^	ENT	0.85	15.61^***^	R/E	0.34	2.47^*^	REM	0.24	1.83	REM	0.21	1.53
FemF^b^	R/E	0.25	4.31^***^	ENT	0.63	4.78^***^	ENT	0.75	6.18^***^	ENT	0.49	3.61^**^
**AUSTRIA – ONLINE**												
MascM^a^	ENT	0.76	9.76^***^	R/E	0.48	4.17^***^	R/E	0.30	2.47^*^	R/E	0.49	8.37^***^
MascF^a^	REM	0.19	1.80	ENT	0.67	4.95^***^	ENT	0.86	9.21^***^	ENT	0.81	12.39^***^
FemM^b^	R/E	0.37	3.84^***^	REM	0.17	1.18	REM	0.23	1.72	R/E	0.32	4.45^***^
FemF^b^	ENT	0.72	8.70^***^	ENT	0.69	7.35^***^	ENT	0.80	7.35^***^	ENT	0.79	11.45^***^

*MascM, masculinity in reference to men; mascF, masculinity in reference to women; femM, femininity in reference to men; femF, femininity in reference to women*.

a *Tested as predictors of masculinity in reference to the entire population*.

b *Tested as predictors of femininity in reference to the entire population*.

* *p < 0.05*,

** *p < 0.01*,

*** *p < 0.001, ENT, variable entered by procedure; REM, variable removed by procedure; R/E, removal yields better model, but entry of variable also admissible. For ENT variables the β- and t-values for sole entry are reported, for REM or R/E variables the β- and t-values as if entered are reported. Reference groups were determined from ENT variables and marked light gray*.

### Does the sex role self-concept relate to sex hormone levels?

Sex hormone levels were available from 35 men, 18 follicular women, 21 luteal women, and 33 OC users in the US undergraduate sample, as well as from 39 men, 19 follicular, and 14 luteal women in the Austrian undergraduate sample. Means and Standard deviations for each group are displayed in Table 4.

**Table 4:** **Hormone levels (*****M*** **±** ***SD*****)**.

	**Testosterone**	**Estradiol**	**Progesterone**
**CALIFORNIA – PP**			
Men (*n* = 35)	156.93±50.47	2.34±0.94^b^^**^	73.00±50.47^b^^***^
Follicular (*n* = 18)	74.44±27.93^a^^***^	2.64±0.89	76.94±37.36^b^^**^
Luteal (*n* = 21)	64.89±20.39^a^^***^	3.14±0.98	148.58±109.28
OC (*n* = 33)	49.78±27.77^a^^***^	2.60±1.01	
**AUSTRIA – PP**			
Men (*n* = 39)	112.87±47.12	2.63±0.80^b^^***^	94.21±97.98^b^^***^
Follicular (*n* = 19)	46.76±19.17^a^^***^	2.51±0.83^b^^***^	71.38±74.61^b^^***^
Luteal (*n* = 14)	53.12±27.27^a^^***^	2.83±1.48	385.68±382.57

a *Significantly lower than in men*.

b *Significantly lower than in luteal women*.

** *p < 0.01*,

*** *p < 0.001*.

#### All participants

In both samples, testosterone correlated positively with all masculinity ratings (all *r* > 0.49, all *p* < 0.001) and negatively with all femininity ratings (all *r* < -0.48, all *p* < 0.001). In the US undergraduate sample, estrogen predicted masculinity ratings negatively (both *r* < -0.19, all *p* < 0.05) and femininity ratings positively (both *r* > 0.18, both *p* < 0.06) in reference to men and women, but not the ratings in reference to the entire population (both |*r*|< 0.16, both *p* > 0.10). These correlations with estrogen did not reach significance in the Austrian sample. In the US undergraduate sample, progesterone predicted all masculinity ratings negatively (all *r* < -0.24, all *p* < 0.05) and all femininity ratings positively (all *r* > 0.34, all *p* < 0.01). In the Austrian undergraduate sample, these correlations with progesterone only reached significance for ratings in reference to men (masc: *r* = −0.27, *p* < 0.05; fem: *r* = 0.24, *p* < 0.05).

In the single group analyses, we found contrary results in the US and Austrian sample.

#### Men

Estrogen predicted masculinity ratings in reference to the entire population positively (*r* = 0.38, *p* < 0.05) and femininity ratings in reference to the entire population and to men negatively (both *r* < -0.38, both *p* < 0.05) in US men. The more estrogen, the more masculine and the less feminine US men rate themselves. However, estrogen predicted masculinity ratings in reference to women negatively (*r* = -0.36, *p* < 0.05), while femininity ratings were unaffected by estrogen in Austrian men. The more estrogen, the less masculine Austrian men they rate themselves. Progesterone and testosterone did not affect sex role ratings in men in any sample (all *r* < 0.17, all *p* > 0.29).

#### Follicular women

In follicular women of the US sample, only femininity ratings in reference to men showed an interrelation with sex hormone levels. They were positively predicted by both progesterone (*r* = 0.50, *p* < 0.05) and testosterone (*r* = 0.59, *p* < 0.01), but unrelated to estrogen (*r* = 0.18, *p* = 0.48). The more progesterone and testosterone, the more feminine US follicular women rated themselves in reference to men. In follicular women of the Austrian sample, testosterone and progesterone levels were not significantly related to sex role ratings. However, estrogen levels affected follicular women's sex role ratings in reference to other women. Masculinity ratings were positively predicted by estrogen levels (*r* = 0.49, *p* < 0.05), while femininity ratings were negatively predicted by estrogen levels (*r* = -0.59, *p* < 0.01). The more estrogen, the more masculine and the less feminine Austrian follicular women rated themselves in reference to other women.

#### Luteal women

In luteal women of the US sample, we found estrogen to positively predict femininity ratings (significantly in reference to women: *r* = 0.46, *p* < 0.05; by trend in reference to men: *r* = 0.37, *p* = 0.09). The more estrogen, the more feminine US luteal women rated themselves. In luteal women of the Austrian sample, no relationship between sex role ratings and sex hormone levels was observed.

Sex hormone levels and sex role were unrelated in OC users of the California PP sample (all |*r*|< 0.15, all *p* > 0.30).

### Do culture, sex or hormonal status affect the factorial structure of the sex role self-concept

Results of Principal Component Analyses over the six sex role ratings in the three samples are summarized in Table 5. In the Northern American sample, sex role seems to comprise a one factor structure in men and OC users with masculinity and femininity comprising opposite poles of the same construct, but a 2 factor structure in naturally cycling women with masculinity and femininity items loading high on different factors. In both Middle European samples, sex role seems to comprise a 2 factor structure in men, with masculinity and femininity ratings loading high on different factors and a 1 factor structure in OC users with masculinity and femininity comprising opposite poles of the same construct. In follicular women of the middle European sample, a 2 factorial structure was obtained. However, masculinity and femininity ratings do not completely load on different factors. Reference group seems to be a second determinant of the factors. Luteal women display a one factor solution like OC-users in the PP undergraduate sample and a 2 factorial solution like men in the online sample.

**Table 5:** **Varimax rotated solution of principal components analysis with open number of factors**.

	**Men**		**Follicular**		**Luteal**		**OC**	
	**Factor1**	**Factor2**	**Factor1**	**Factor2**	**Factor1**	**Factor2**	**Factor1**	**Factor2**
**CALIFORNIA – PP**								
mascM	−0.85			0.92	0.85	0.89		
MascF	−0.74			0.82	0.81	0.86		
mascALL	−0.80			0.94	0.78	0.92		
FemM	0.88		0.91		0.81	−0.74		
FemF	0.81		0.84		0.92	−0.87		
femALL	0.89		0.91		0.95	−0.83		
Variance	68.80	10.25	48.25	35.17	62.11	19.34	73.00	11.82
PCA solution	1 component		2 components		2 components		1 component	
**AUSTRIA – PP**								
mascM	0.83		0.94		0.65	0.72		
MascF	0.80			−0.68	0.83	0.82		
mascALL	0.86		0.76		0.88	0.80		
FemM		0.92	−0.72		−0.70	−0.57		
FemF		0.64		0.92	−0.81	−0.67		
femALL		0.94		0.78	−0.83	−0.70		
Variance	58.66	18.87	53.34	21.35	62.03	16.51	51.35	16.49
PCA solution	2 components		2 components		1 component		1 component	
**AUSTRIA – ONLINE**								
MascM	0.91			0.93	0.80	0.77		
mascF	0.84		−0.62	0.52	0.94	0.85		
mascALL	0.88			0.87	0.95	0.88		
FemM		0.91	0.93			0.82	−0.75	
femF		0.76	0.67			0.86	−0.80	
FemALL		0.87	0.89			0.78	−0.83	
Variance	60.88	19.91	50.22	26.63	57.86	23.30	66.11	15.68
PCA solution	2 components		2 components		2 components		1 component	

*MascM, masculinity in reference to men; mascF, masculinity in reference to women; mascALL, masculinity in reference to the entire population; femM, femininity in reference to men; femF, femininity in reference to women; femALL, femininity in reference to the entire population*.

### Typology

Table 6 summarizes the frequency of each sex role type for men, follicular women, luteal women, and OC users among the three samples. In the California-PP undergraduate sample and the Austrian online sample drawn from the general population, there was a significantly higher number of indifferent participants relative to androgynous participants among men compared to the female groups (both χ^2^ > 4.36, *p* < 0.05), while there was a trend toward a higher number of indifferent participants relative to androgynous participants among women compared to men in the Austrian undergraduate sample (χ^2^ = 3.12, *p* = 0.08). The absolute number of indifferent and androgynous participants did not differ between men and women in sample (a) (both χ^2^ < 2.62, both *p* > 0.10). In sample (b) there was a significantly higher number of women than men among indifferent participants (χ^2^ = 4.84, *p* < 0.05), but not among androgynous participants (χ^2^ = 0.76, *p* = 0.38). In sample (c) there were by trend more indifferent and less androgynous participants among men compared to women (both χ^2^ > 3.49, both *p* = 0.06). Furthermore, there was a significantly higher number (χ^2^ = 8.57, *p* < 0.01) of flips among women (female → male) compared to men (male → female) in sample (c), but not in samples (a) and (b) (both χ^2^ < 0.52, both *p* > 0.47).

**Table 6:** **Frequency [%] of Typologies among men and women in the three samples**.

	**Masc**	**Fem**	**(a) California – PP**				**(b) Austria – PP**				**(c) Austria – online**			
			**Men**	**Foll**.	**Luteal**	**OC**	**Men**	**Foll**.	**Luteal**	**OC**	**Men**	**Foll**.	**Luteal**	**OC**
Indiff.	low	low	16.2	11.1	9.1	4.0	10.4	22.2	25.8	18.6	21.9	19.4	14.3	7.3
Male	high	low	78.4	5.6	0.0	4.0	79.2	2.8	3.2	4.7	76.7	3.2	14.3	9.8
Female	low	high	5.4	72.2	81.8	88.0	4.2	69.4	67.7	74.4	0.0	71.0	64.3	74.4
Androgyn	high	High	0.0	11.1	9.1	4.0	6.3	5.6	3.2	2.3	1.4	6.5	7.1	8.5

*Foll., follicular; OC, Oral contraceptive; indiff., indifferent; PP, paper and pencil; masc, masculinity; fem, femininity*.

### Characteristics of the sex role self-concept

Table 7 summarizes which features are commonly named by participants to explain their masculinity and femininity ratings. The inter-rater agreement was 95%. Only categories on which both raters agreed are listed. In both samples, more women than men gave explanations for their sex role ratings. While Northern American participants named mostly activities and the importance of beauty, fashion and body care as determinants for their sex role ratings, middle European participants referred predominantly to personality traits and only secondarily to activities and the importance of beauty, fashion and body care.

**Table 7:** **Percentage of participants naming features as explanation for their sex role ratings that were categorized to the categories on the left**.

	**Men**	**Follicular**	**Luteal**	**OC users**	**Total**
CALIFORNIA – PP					
Face and body	0	11	0	0	2
Beauty care	0	28	18	6	10
Activities	5	22	23	12	13
Social contacts	0	17	9	0	4
Personality	5	17	9	3	7
Emotion	3	0	0	3	2
Cognition	0	0	0	3	1
AUSTRIA – ONLINE					
Face and body	4	6	0	7	5
Beauty care	4	12	22	7	9
Activities	7	6	25	7	10
Social contacts	3	6	9	9	6
Personality	18	16	22	21	19
Emotion	4	6	3	0	3
Cognition	1	9	0	2	3

## Discussion

The present study assessed self-rated masculinity and femininity in relation to hormonal status and sex hormone levels in participants from different cultures and different educational and socioeconomic status. Influence of culture and hormonal status were investigated on absolute sex role ratings, factorial structure of sex role ratings, reference group and aspects participants rated as relevant to their sex role orientation.

### Men compare themselves to men, women to women, but not during the follicular phase

Menstrual cycle does not affect self-perceived masculinity or femininity, even though it has been demonstrated to significantly modulate cognitive sex differences (Hampson, 1990; Hausmann et al., 2000; Rosenberg and Park, 2002). Also only minor differences in the factorial structure of sex role were observed between the cycle phases, as shall be discussed in the following. However, menstrual cycle phase seems to affect which reference group women use when determining their masculinity and femininity scores.

Regression analyses revealed that across cultures, men compare themselves more to men, even when explicitly asked to make comparisons in reference to the entire population. As masculinity ratings in reference to men are naturally lower than masculinity ratings in reference to women, ratings in reference to the entire population may underestimate men's masculinity and/or vice versa overestimate their femininity. Likewise, naturally cycling women during their luteal phase as well as OC users compare themselves to other women and their ratings in reference to the entire population may therefore, underestimate their femininity and/or overestimate their masculinity.

During the follicular cycle phase, however, naturally cycling women, show a tendency to compare themselves to men (US sample) or men and women about equally (Austrian samples). Consequently, their self-ratings in reference to the entire population may be most accurate. Interestingly, in the Austrian samples, follicular women seem to determine their masculinity in reference to men and their femininity in reference to women. This was not only reflected in the regression analyses, but in the factorial structure of their sex role ratings, suggesting two different bipolar dimensions for ratings in reference to men and ratings in reference to women. This shift in reference group across the menstrual cycle is in good accordance with the shifts from increased spatial (typically male-dominated) to increased verbal (typically female dominated) abilities from the follicular to the luteal phase (e.g., Hampson, 1990; Hausmann et al., 2000; Rosenberg and Park, 2002). A similar shift in absolute sex role ratings might be observable in a within-subjects design to compare the cycle phases rather than the between-subjects design employed in the present study. Consequently, menstrual cycle phase should be taken into account when assessing a person's sex role orientation.

### OC-use alters sex role orientation and its factorial structure

Hormonal contraceptive users rated themselves as significantly more feminine and significantly less masculine than naturally cycling women independent of culture. This effect is striking since both masculinizing and feminizing effects of hormonal contraceptives have been reported in the cognitive domain. However, even though OC users score better in spatial tasks than naturally cycling women (Wright and Badia, 1999; Wharton et al., 2008) and show masculinized brain activation patterns during number processing (Pletzer et al., 2014), they do perceive themselves as less masculine than naturally cycling women irrespective of their cycle phase. The simultaneous perception of reduced masculinity and enhanced femininity leads to a one-factorial structure of sex role in OC-users as opposed to the two-factorial structure mostly observed in naturally cycling women. Consequently, more female and less androgynous, indifferent or flipped to male sex types were observed in OC-users as compared to naturally cycling women. These results are particularly important as they could be replicated across three different samples, from different cultures, different educational, and socioeconomic status and different mode of data collection. It remains to be resolved, how the synthetic progestins and ethinyl-estradiol contained in OC mimic endogenous sex hormone actions to cause this shift in sex role orientation.

### The relationship between estrogen and sex role orientation depends on its factorial structure

Correlations of masculinity and femininity ratings with sex hormone levels as observed over all participants are in accordance with sex differences in sex role ratings. Male sex and high testosterone favor high masculinity and low femininity ratings, while female sex and high estrogen and/or progesterone favor high femininity and low masculinity ratings. Importantly, while testosterone and progesterone seem to be more responsible for the modulation of sex role ratings across groups, estrogen seems to modulate sex role ratings within the different hormonal status groups. However, these within-group results are puzzling at first sight. As the differences between the US and Austrian sample in correlations between sex hormones and sex role ratings somehow resemble the differential factorial structure of sex role in men and women between the US and Austrian undergraduate samples, these shall be discussed in parallel.

In the US, undergraduate men perceive masculinity and femininity as two bipolar ends of one dimension and their estrogen levels are positively related to masculinity (and negatively to femininity). In Austria, undergraduate men perceive masculinity and femininity as two different dimensions and their estrogen levels are negatively related to masculinity (but not to femininity).

In the US, undergraduate naturally cycling women perceive masculinity and femininity as two different dimensions and their (luteal phase) estrogen levels are positively related to femininity (but not to masculinity). In Austria, undergraduate naturally cycling women perceive masculinity and femininity as bipolar ends of one dimension and their (follicular phase) estrogen levels are negatively related to femininity (and positively to masculinity).

A one-factorial structure seems to correspond to a paradoxically reversed relationship between the “female” sex hormone estrogen and sex role, while a two-factorial structure corresponds to the expected relationship between estrogen and masculinity in men/femininity in women. One psychological mechanism explaining this correspondence could be compensation. If participants assume that being highly feminine makes them less masculine, and vice versa, high estrogen men, who are presumably more feminine, or low estrogen women, who are presumably more masculine, may—in an attempt for socially desirable self-presentation—overcompensate for this fact by rating themselves as more masculine or more feminine, respectively. On the contrary, if participants acknowledge that one can be feminine without having to be less masculine or vice versa, their self-ratings may be more reflective of their actual sex role orientation. However, our findings may also be interpreted in the light of previous research demonstrating that the relationship between sex hormones and behavior is not always linear and may differ between men and women (Tan, 2012).

### Culture reverses sex differences in the factorial structure of sex role orientation

Furthermore, the sex difference in factorial structure was reversed between the Austrian and US undergraduate samples. In the US sample, men appear to think they can only be feminine when not being masculine, while women incorporate both characteristics. In the Austrian sample however, women appear to think they can only be masculine when not being feminine, while men incorporate both characteristics. These data may reflect a cultural tendency to favor masculine characteristics among US undergraduates as opposed to a cultural tendency to favor feminine characteristics among Austrian undergraduates. This results in a lack of androgynous, but high number of indifferent men in the US sample as opposed to a low number of androgynous, but high number of indifferent women in the Austrian sample. Note however that factorial structures in the Austrian Online sample, which was drawn from a more general population, are more similar to the US data, suggesting a role of education and socioeconomic status in these tendencies. Furthermore, in neither sample did we observe such high numbers of androgynous participants as in Bem's original study (Bem, 1974).

To evaluate, whether these cultural differences may be attributed in culturally different perception of the sex role concept, US undergraduate and Austrian online participants were asked to explain their sex role ratings. Note that differences in factorial structure were not as extreme between these two samples as between the two undergraduate populations. Nevertheless, curious differences were found between the explanatory variables reported in the two samples. While middle European participants rely most strongly on personality measures to explain their sex role ratings, American participants rely more strongly on activities and interests on the one hand and appearance on the other hand (beauty care). Consequently, while personality is an important dimension of sex role orientation as incorporated in social role theory (Eagly, 2013) and may favor a two-factorial structure, this finding provides support for previous criticism on measures such as the PAQ and BEM, which involve only personality items. Especially, approaches to determine sex role orientation across cultures and seeking to overcome problems with self-reflection and social desirability could profit from including additional dimensions that are more continuous and can be expressed to varying degrees in both men and women.

In summary, we found with respect to our research questions, that neither culture nor menstrual cycle affected sex role ratings per se, while OC use did rate themselves more feminine and less masculine than naturally cycling women. However, culture and hormonal status interactively affected whether participants compared themselves more to men or women in their sex role ratings. Also, the impact of sex hormones on sex role orientation was not only modulated by sex and hormonal status, but also by culture. Both culture and hormonal status are also important determinants of the factorial structure of sex role orientation and culture influences which characteristics are viewed as important for sex role orientations by participants themselves. These findings have important implications for the assessment of sex role orientation in different cultures.

Altogether our data are in line with previous psychobiological approaches, suggesting that sex role orientation as well as the sex role self-concept are shaped by both biological mechanisms, such as sex hormones as well as social/cultural influences (Halpern and Tan, 2001).

### Conflict of interest statement

The authors declare that the research was conducted in the absence of any commercial or financial relationships that could be construed as a potential conflict of interest.

## Footnotes

^1^This assumption was confirmed by significant results of a Kolmogorov–Smirnov Test for normality for all six variables.
